# Depressive symptom trajectories over a 6-year period following myocardial infarction: predictive function of cognitive appraisal and coping

**DOI:** 10.1007/s10865-015-9681-y

**Published:** 2015-09-30

**Authors:** Aleksandra Kroemeke

**Affiliations:** Department of Psychology, University of Social Sciences and Humanities, Chodakowska Street 19/31, 03-815 Warsaw, Poland

**Keywords:** Depression, Coping, Cognitive appraisal, Myocardial infarction, Longitudinal study, Growth mixture modeling

## Abstract

The association between distinct patterns of depression and coping variables in myocardial infarction (MI) survivors over the long-term is unclear. The study aims to evaluate depressive trajectories and their covariates, including coping and cognitive appraisal, following MI over a period of 6 years. Depressive symptoms were assessed in 200 patients a few days after the first MI, and 1 month, 6 months and 6 years later. Cognitive appraisal and coping were assessed during the first three time points. Three latent depressive trajectories were identified: chronic (high; increasing then decreasing; *n* = 49), rising (moderate; decreasing then increasing; *n* = 121) and low (low; decreasing then stabilizing; *n* = 30). The chronic trajectory was associated with higher negative appraisal and emotion-focused coping. The findings clarify the long-term longitudinal trajectories of post-MI depressive symptoms and their association with coping variables, revealing the unfavorable impact of negative cognition and palliative coping.

**Electronic supplementary material:**

The online version of this article (doi:10.1007/s10865-015-9681-y) contains supplementary material, which is available to authorized users.

## Introduction

Depression is more common in medical patients than in the general population (Katon, 2003). Its frequency in patients after myocardial infarction (MI) is more than twice that in the healthy population (Thombs et al., 2006). The extent of post-MI depression and its predictors have been frequently studied (Myers et al., 2012; Thombs et al., 2006); however, only few studies have focused on changes in depressive symptoms over time. Moreover, the results of majority of existing studies were based on statistical averages for an entire group of patients (Delisle et al., 2012; Grace et al., 2005; Hanssen et al., 2009). However, it is unlikely that all post-MI patients experience changes in depressive symptoms in the same manner. Indeed, the coping process associated with MI and emotional adaptation to MI varies with the individual and depends on multiple factors. Thus, the present study aims to identify homogeneous subpopulations with different depression trajectories within a heterogeneous sample of MI patients and to evaluate the effects of coping variables on different patterns of depression.

### Depression after myocardial infarction

Based on meta-analysis data (Thombs et al., 2006) about 20 % of post-MI patients experience symptoms of clinical depression during hospitalization, and this number increases significantly post-hospitalization. Subclinical depressive symptoms after MI are likely to be even more prevalent. Post-MI depression is connected with physical limitations (de Jonge et al., 2006), cardiac complications and re-hospitalization (Myers et al., 2012), as well as an increased probability of subsequent MI and patient mortality (Larsen et al., 2014). In addition, depressed patients participate in cardiological rehabilitation and modify their lifestyles less frequently (Benyamini et al., 2013; Myers et al., 2012), which indirectly impacts their health and prognosis.

Relatively few studies have examined changes in depressive symptoms over time after MI, and those that have, have produced inconclusive results. Some studies suggest that the level of post-MI depression is stable within 3 months (Bennett et al., 2002), 6 months (Delisle et al., 2012), 1 year (McGee et al., 2006), and even longer (up to 18 months; Hanssen et al., 2009), post-MI. Conversely, other studies have reported decreases in depressive symptoms in comparable timeframes (Grace et al., 2005; Yohannes et al., 2010). The inconsistencies may stem from the fact that these studies were based on mean depressive symptoms scores for the entire sample, according to a variable-centered perspective (Laursen & Hoff, 2006), which means that the presence of homogeneous subpopulations with different depression levels within a heterogeneous sample of cardiac patients were not considered.

It is likely that not everyone adjusts to MI the same way and that depression trajectories are different from patient to patient. The basis of this premise is congruent with a transaction model of stress and coping (Lazarus & Folkman, 1984), according to which, adjusting to a difficult situation depends on personal and social antecedents, cognitive appraisal of a stressful event, and coping. It can be expected that patients would experience higher or lower post-MI depressive symptoms depending on their coping resources, event appraisal and coping efforts. Therefore, it is justified to identify homogeneous subpopulations of patients with different patterns of change in post-MI depressive symptoms.

### Distinct trajectories of changes in depressive symptoms

The interest in heterogeneous trajectories of depression following MI is increasing. In a study of patients 1 year after MI, Kaptein et al. (2006) indentified five different longitudinal patterns of depressive symptoms: none, mild, moderate and increasing, significant but decreasing, and significant and increasing. Martens et al. (2008) found four stable trajectories differing in symptoms intensity in 1-year-post-MI patients (mild, none, moderate and severe). In contrast, Murphy et al. (2008) reported two quadratic trajectories of depression over a similar timeframe in women after MI or before cardiac surgery. In a recent population-based prospective study covering a period of 6 years prior to MI to 4 years after MI, Galatzer-Levy and Bonanno (2014) identified four patterns of depressive symptoms with the largest resilient class, and smaller classes characterized by chronic, emerging and improved depression. These findings are compatible with prototypical mental health outcome trajectories following potentially traumatic life events (Bonanno, 2004). Nonetheless, there is still little data on variation in trajectories of depressive symptoms among MI survivors, especially over the long-term.

### Predictors of belonging to depression trajectories

In a practical sense, it is not sufficient to merely identify the trajectory of changes. More important is to study the determinants and correlates of these trajectories. According to stress and coping theory, distinct patterns of change in stress outcomes can be ascribed to two crucial coping process variables—cognitive appraisal and coping strategies (Lazarus & Folkman, 1984).

Cognitive appraisal refers to the evaluation of the significance of an event for one’s well-being, which may be interpreted as threatening, harmful or challenging (Lazarus & Folkman, 1984). A recent study by Bonanno et al. (2012) revealed that challenge appraisal predicted depressive trajectory following spinal cord injury, indicating higher challenge appraisal in the subgroup with low symptoms. Relatedly, Chilcot et al. (2013) found that illness perceptions were associated with distinct patterns of depressive symptoms among dialysis patients. Patients with low symptoms had more positive and adaptive illness perceptions than patients from the high-reducing and moderate-increasing subgroups.

Coping refers to dynamically fluctuating thoughts and behaviors undertaken to manage a stressful transaction (Lazarus & Folkman, 1984). It comprises problem-focused behaviors, as well as strategies directed at regulating emotional reactions or avoidance. However, data on coping are not as consistent as those for cognitive appraisal. Specifically, some studies have found support for the prediction of inclusion in a particular depressive trajectory class based on coping strategies (Bonanno et al., 2012; Lambert et al., 2012), whereas others have not (Donovan et al., 2014). Bonanno et al. (2012) found that patients with stable low depression were more likely to cope using problem-focused strategies and less likely to use emotion-focused strategies or avoidance, compared with patients with chronic depression. Lambert et al. (2012) merely confirmed the relationship between avoidance and depressive trajectory. Moreover, the findings of Bonanno et al. (2012) suggest that the relationships can be curvilinear, and that patients with delayed symptoms may be the most dysfunctional.

Taken together, the evidence suggests that two crucial coping process variables, cognitive appraisal and coping, may constitute potential variables explaining membership in longitudinal pattern of depression. Their role in predicting changes in stress outcomes, however, still requires clarification. Data on the role of cognitive appraisal as an antecedent of different change trajectories is particularly scarce. Coping is analyzed more often, although the findings are inconsistent. No studies were found that have investigated coping process variables as predictors of longitudinal depressive trajectories among MI survivors.

### The present study

The primary aim of this study was to identify the intra-individual change trajectories of depressive symptoms among MI survivors over a period of 6 years. As can best be determined, this study is the first to examine patterns of change in post-MI depressive symptoms over such a long-term. Based on Bonanno’s (2004) theorizing and evidence from studies reviewed here, it was hypothesized that three or four different trajectories of depressive symptoms would be found. Specifically, it was expected to find classes with stable post-MI depressive symptoms: low, moderate or high (Hypothesis 1a). It was assumed that some people would not experience post-MI depression, and others would show moderate or high symptoms which would persist over longer periods of time and become chronic. Furthermore, given the extended follow-up time, classes with changes in post-MI depressive symptoms—either increasing or decreasing—were expected (Hypothesis 1b). For some cardiac patients, MI may be a single-incident event which may produce short perturbations in normal adjustment, and then a gradual return to pre-cardiac event functioning may ensue. Others may experience an exacerbation of existing symptoms over time.

The second aim was to evaluate whether cognitive appraisal and coping strategies help determine a particular trajectory. To the best of knowledge, this study is the first to examine crucial coping process variables as predictors of depressive trajectory in MI survivors. To date, most studies have focused on socio-demographic and health-related characteristics of different post-MI depressive patterns (Galatzer-Levy & Bonanno, 2014; Kaptein et al., 2006; Martens et al., 2008; Murphy et al., 2008). Based on stress and coping theory (Lazarus & Folkman, 1984), it was hypothesized that cognitive appraisal and coping strategies would be linked to different patterns of post-MI depressive symptoms. Specifically, it was expected that high challenge and low threat/harm appraisal would predict inclusion in classes with lower post-MI depressive symptoms compared to classes with higher scores (Hypothesis 2a). Furthermore, high problem-focused and low emotion- and avoidance-focused coping were assumed to predict inclusion in subgroups with lower symptoms compared to higher ones (Hypothesis 2b).

## Methods

### Participants and procedure

Two hundred Polish cardiac patients (age 53.73 ± 7.26 years; 70.5 % men) participated in the study and were assessed four times: a few days after the first MI (T1), and 1 month (T2; *n* = 180), 6 months (T3; *n* = 174) and 6 years (T4; *n* = 111) later. Inclusion criteria were: first myocardial infarction without mechanical complications (such as a left ventricular free wall rupture, ventricular septal defect, acute mitral insufficiency, papillary muscle rupture), absence of serious co-morbidities (cancer, neurological and psychiatric disorders), and age ≤65 years. T1 assessment was conducted in the hospital, T2 and T3 at the respondent’s home, while at T4, questionnaires were distributed by mail (only to 174 persons who participated in the study at T3) and were returned in closed pre-stamped envelopes (the response rate was 63.8 %; 15 participants refused to continue in the study, 7 died and contact was lost with 67). Most participants (85 % at T1 and 83 % at T4) were in a stable relationship (either married or cohabiting), and declared at least average socioeconomic status (87 % at T1 and 80.5 % at T4). Of the participants, 43 % had at least a secondary education, 54 % were employed before MI, and 37 % were employed 6 years later. The participants typically fell under two classes of heart failure according to the New York Heart Association (1994) functional classification system: Class I (49 % at T2 and 47 % at T4) and II (49 % at T2 and 41 % at T4). Moreover, 6 years after MI, the presence of critical life events over the past year were controlled for using the Recent Life Changes Questionnaire RLCQ (Miller & Rahe, 1997). The occurrence of 40 selected essential life changes within the last year related to health, work, home and family, personal and social, as well as financial domains was assessed on a two-point scale from 0 (not occurred) to 1 (occurred); *M* = 7.37, *SD* = 3.64, range 0–19. Cronbach’s α coefficient was .63.

All questionnaires were completed after written informed consent was obtained from the participants, and the study was approved by the University of Social Sciences and Humanities ethics committee in Warsaw. Participation was voluntary. During the first three time points, participants completed questionnaires measuring cognitive appraisal, coping and depressive symptoms. At T4, only depressive symptoms were assessed.

Sample attrition analyses (using binomial logistic regression) indicated that the completers and non-completers did not differ in terms of socio-demographic (age, sex, education, marital status, children, economic status or employment), health-related (NYHA, co-morbidities, medication use) or major study variables (depressive symptoms, cognitive appraisal or problem- and emotion-focused coping) at any timepoint, except avoidance-focused coping at T3 (*B* = −0.10, *SE* = 0.04, *p* = .009).

### Measures

*Depressive symptoms* were assessed with the Beck Depression Inventory (BDI) (Beck et al., 1988). Internal consistency coefficients of the score of this scale ranged from α = .86 at T1 and 4 to α = .89 at T3.

*Cognitive appraisal* was measured with the Stress Appraisal Questionnaire (Wrześniewski & Włodarczyk, 2001) that assessed the negative (threat and harm/loss, 10 items, e.g., *This situation was terrifying*; α from .89 at T1 to .93 at T3; total score: 10–40) and positive cognitive appraisals (challenge, 7 items, e.g., *This situation was mobilizing*; α from .74 at T1 to .78 at T3; total score: 7–28). Respondents rated the extent to which they perceived a specific stress situation (MI and somatic health) on a four-point scale from 1 (not at all true) to 4 (completely true). Inter-scale correlations were non-significant.

*Coping* was assessed with the situation-specific Coping Inventory for Stressful Situation (CISS-S) developed by Endler and Parker (1994) to measure problem-focused (7 items, e.g., *I focus on the problem and on how to solve it*; α from .75 at T3 to .79 at T2), emotion-focused (7 items, e.g., *I am worried*; α from .77 at T2 to .81 at T1 and 3) and avoidance-focused coping strategies (7 items, e.g., *Phone a friend*; α from .65 at T3 to .70 at T1). Participants rated the extent to which they undertook certain behaviors in a stressful situation (in relation to the MI and somatic health) on a five-point scale (total score: 7–35 for each of the subscales). Inter-scale correlations were significant, but weak.

### Statistical analysis

Descriptive statistics were computed using SPSS 22. Missing values were less than 1.5 % at T1, 10 % at T2, 13 % at T3, and 45 % (depression only) at T4. Except for avoidance measured at T3, no systematic attrition was observed. Moreover, Little’s MCAR test pointed to random missingness (*p* = .66). According to Hedeker and Gibbons’ (1997) dropouts diagnostic recommendations, patterns of change over time in avoidance in the completers and non-completers were tested. No interaction effect (time × group) was found (Wilk’s lambda, *F* = 2.595; *df* = 2.170, *p* = .078), thus, a similar trajectory of avoidance in completers and non-completers emerged. As it is recommended (Graham, 2009), missing data was multiple imputed, which is one of the best methods currently available to dealing with missingness. Depressive symptoms were imputed using growth mixture modeling. Missingness of the other major variables was multiple imputed in SPSS using all analyzed variables. Consistent with suggestions by Hardt et al. (2012), no more variables than 1/3 of completers number were included into imputation. The comparison between original (*N* = 111) and imputed (*N* = 200) data-sets revealed no significant sample differences in analyzed variables. Thus, the results from the imputed database were reported.

To identify heterogeneous trajectories of depressive symptoms among MI patients, growth mixture modeling (GMM) (Duncan et al., 2011) was conducted using Mplus statistical package ver. 7 (Muthén & Muthén, 1998). The maximum likelihood with robust standard errors was used as estimator (Muthén & Muthén, 1998). Factor loadings corresponded directly to the time interval (T1 was set to 0 and T4 to 12). Both linear and quadratic slopes were estimated. The determination of the appropriate latent class solution was based on: (a) the Bayesian Information Criterion (BIC); (b) the Akaike Information Criterion (AIC); (c) the Bootstrap Likelihood Ratio Test (BLRT); (d) the Vuong-Lo-Mendell-Rubin Likelihood Ratio Test (VLMRLRT); (e) entropy value; and (f) practical usefulness of the latent trajectory classes (Duncan et al., 2011; Jung & Wickrama, 2008). The model with the lower BIC and AIC values, greater entropy value (closer to 1), and significant BLRT and VLMRLRT tests (*p*s < .05 indicated that the estimated model is preferable over a model with one fewer class) indicated good fitting (Duncan et al., 2011; Jung & Wickrama, 2008). Results were replicated to avoid local solutions (cf. Jung & Wickrama, 2008).

After identifying depressive symptoms trajectories, the antecedents of each depressive latent class were tested with SPSS 22. First, multinomial logistic regression analysis (MLR) was conducted to determine the relationship between background predictors: socio-demographical and health-related variables and inclusion in latent trajectory groups. To test the hypothesis, i.e. how coping process variables (cognitive appraisal and coping strategies) change within each depressive trajectory, several series of multivariate repeated measures of analyses of covariance (MANCOVA) were conducted with depressive latent classes as the between-subject variable and time as the within-subject variable. Background variables significantly related with dependent variables were included as covariates.

## Results

Descriptive statistics and average rates of change over time are presented in Table 1. Overall, the participants reported lower levels of depressive symptoms 1 month after MI (T2), which remained unchanged in the follow-ups (T2–T4). Except for positive appraisal, the level of all other variables decreased within 6 months after MI.

**Table 1:** Descriptive statistics (*mean* *±* *SD*; minimum–maximum) and MANOVA results for major study variables (*N* = 200)

Variables	Few days after MI	1 month after MI	6 months after MI	6 years after MI	MANOVA^a^ partial eta^2^	Bonferroni’s test
	T1	T2	T3	T4		
Depressive symptoms	10.57 ± 8.60	8.88 ± 7.13	7.92 ± 7.48	8.50 ± 6.50	10.42***	T1 > T2, 3, 4
	0–44	0–34	0–36	0–31	.14	
Negative appraisal	28.22 ± 7.21	25.34 ± 7.78	22.85 ± 8.47		49.20***	T1 > T2 > T3
	10–40	10–40	10–40		.33	
Positive appraisal	21.87 ± 3.93	22.31 ± 3.75	22.23 ± 4.02		1.29	
	9–28	9–28	9–28			
Problem coping	27.42 ± 5.38	26.98 ± 5.48	25.64 ± 5.55		11.16***	T1, 2 > T3
	7–35	9–35	8–35		.12	
Emotion coping	18.04 ± 6.22	16.00 ± 5.59	15.68 ± 5.82		19.72***	T1 > T2,3
	6–30	6–30	6–30		.17	
Avoidance coping	14.63 ± 4.97	13.97 ± 4.56	13.44 ± 4.53		7.51**	T1 > T3
	5–25	5–25	5–25		.07	

** *p* < .01; *** *p* < .001

^a^Wilk’s Lambda *F*; *df* = 3197 for T1–T4 comparisons; *df* = 2198 for T1–T3 comparisons

### Trajectories of depressive symptoms

Table 2 shows the fit indicators for latent class solutions for depressive symptoms in GMM analysis. The AIC value supported the five-trajectory solution; while the entropy value supported the four-trajectory solution. Similarly, the BLRT tests, although the *p* value was not less than .05, might have selected the three-trajectory solution. Finally, the BIC value and the VLMRLRT test supported the three-trajectory solution. Nylund et al. (2007) have suggested that the BIC is the best indicator of the number of class solutions, while both the BLRT and the VLMRLRT correctly identify the number of classes. Accordingly, based on the proportions for the latent classes, the results of testing whether the solutions are local as well as meaningful to the trajectory solutions, the three-class solution was chosen.

**Table 2:** Fit indices for GMMs of depressive symptoms with different latent trajectory classes (*N* = 200)

No. of classes	Log likelihood	Entropy	AIC	BIC	VLMRLRT *p* value	BLRT *p* value	Group sizes (*n*)
2	−2078.824	.727	4197.648	4263.615	.1725	.0000	53/147
3	−2054.141	.729	4162.281	**4251.336**	**.0000**	**.0000**	49/121/30
4	−2038.422	**.752**	4144.844	4256.987	.2397	**.0462**	13/93/39/55
5	−2028.799	.740	**4139.598**	4274.829	.2398	1.0000	92/41/16/14/37

Best fit indicates are in bold

*AIC* Akaike’s Information Criterion, *BIC* Bayesian Information Criterion, *VLMRLRT* Vuong-Lo-Mendel-Rubin Likelihood Ratio Test, *BLRT* Bootstrap Likelihood Ratio Test

**Fig. 1:**
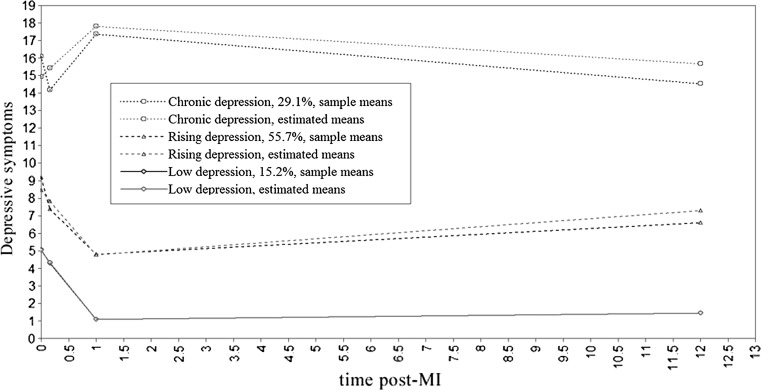
Latent trajectories of depressive symptoms over a 6-year period post-MI

In this solution (Fig. 1), most of the sample (*n* = 121, 55.7 %) fell under a U-shaped curve (intercept = 1.87, *SE* = 0.13, slope = −0.98, *SE* = 0.14, quadratic = 1.08, *SE* = 0.15, *p* < .001) of the initial medium level of symptoms (*M* = 9.24, *SD* = 7.48 at T1 and *M* = 7.45, *SD* = 5.27 at T2), then decreased 6 months after MI (*M* = 5.31, *SD* = 2.99), and subsequently slightly increased (*M* = 7.09, *SD* = 3.75). This symptom pattern was termed *rising*. The second trajectory, termed *chronic* depression (*n* = 49, 29.1 %), also represented a quadratic trend (intercept = 1.78, *SE* = 0.00, *p* > .05, slope = 0.76, *SE* = 0.23, quadratic = −0.86, *SE* = 0.27, *p* < .01) and was characterized generally by high symptoms (*M* = 17.41, *SD* = 9.19 at T1 and *M* = 15.40, *SD* = 8.34 at T2) with an increase 6 months after MI (*M* = 18.61, *SD* = 6.75) and then a slight decrease 6 years later (*M* = 16.01, *SD* = 6.92). In the third class (*n* = 30, 15.2 %; intercept = 1.46, *SE* = 0.07, slope = −1.03, *SE* = 0.00, *p* < .001, quadratic = 1.13, *SE* = 0.00, *p* > .05), termed *low* depression, participants showed relatively low initial levels of depressive symptoms (*M* = 4.77, *SD* = 3.88 at T1 and *M* = 7.45, *SD* = 5.27 at T2) with decreases 6 months later (*M* = 0.97, *SD* = 0.85) and subsequent stability (*M* = 1.87, *SD* = 1.51).

### Sample characteristics of depressive symptoms trajectories

Table 3 shows the characteristics of participants in relation to depressive symptoms trajectories. Multinomial regression models revealed significant associations between inclusion in the trajectory class and participant age, education, employment, NYHA classification, socioeconomic status, and medication use. Participants in the low class were younger, better educated, employed before and after MI, and had better socioeconomic positions compared to the chronic subgroup in particular, but in some cases, also compared with the rising class. They assessed their heart failure better at T3 and 4, and were taking less medication (overall at T4, antidepressants at T2 and 3) compared to members of other classes.

**Table 3:** Sample characteristics of the chronic, rising and low depressive symptoms trajectories using multinomial logistic regression analysis

	Chronic depression *n* = 49 (1)	Rising depression *n* = 121 (2)	Low depression *n* = 30 (3)	*χ* ^2^	*p* value	Trajectory comparisons
A few days after MI (T1)						
Age, mean years (*SD*)	54.31 (7.17)	52.88 (7.21)	49.53 (6.85)	8.14	.017	(1) < (3)
Males (%)	59.2	71.9	83.3	5.61	.061	(1) < (3)
Secondary or lower education level (%)	91.8	92.5	63.4	15.54	<.001	(1), (2) > (3)
Having a partner (%)	40.8	81.0	93.3	4.40	.111	–
Employment, yes (%)	38.8	53.7	80.0	13.44	.001	(1), (2) < (3)
Socioeconomic status (%)						
High	8.2	7.4	3.3	2.67	.615	–
Average	73.5	81.0	86.7			
Low	18.4	11.6	10.0			
Pre-MI antidepressants use (%)	26.5	25.6	23.3	.104	.949	–
1 month after MI (T2)						
Employment, yes (%)	4.4	6.7	6.7	0.31	.856	–
NYHA (%)						
Class 1	37.5	45.5	72.7	3.79	.434	–
Class 2	62.5	52.3	27.3			
Class 3	0.0	2.3	0.0			
Class 4	0.0	0.0	0.0			
Antidepressants use (%)	11.1	3.8	0.0	6.08	.048	(1), (2) > (3)
6 months after MI (T3)						
Employment, yes (%)	15.9	34.0	56.7	13.69	.001	(1) < (2) < (3)
NYHA (%)						
Class 1	31.8	58.0	76.7	22.29	<.001	(1), (2) versus (3)
Class 2	45.5	35.0	23.3			
Class 3	15.9	5.0	0.0			
Class 4	6.8	2.0	0.0			
Antidepressants use (%)	22.7	12.0	0.0	11.54	.003	(1), (2) < (3)
6 years after MI (T4)						
Employment, yes (%)	23.3	31.7	71.4	13.57	.001	(1), (2) < (3)
Socioeconomic status (%)						
High	3.3	6.7	28.6	9.60	.048	(1), (2) versus (3)
Average	80.0	78.3	66.7			
Low	16.7	15.0	4.8			
Daily medication use, mean number (*SD*)	6.87 (3.36)	5.87 (1.97)	4.52 (1.81)	43.55	.004	(1) > (3)
Antidepressants use (%)	10.7	1.7	0.0	5.05	.080	–
NYHA (%)						
Class 1	16.7	46.7	90.5	31.99	<.001	(1), (2) versus (3)
Class 2	60.0	43.3	9.5			
Class 3	20.0	8.3	0.0			
Class 4	3.3	1.7	0.0			
Critical life events, mean number (*SD*)	109.50 (24.18)	124.81 (28.18)	144.64 (17.56)	44.05	.076	(1) < (2) < (3)

*NYHA* New York Heart Association classification

### Changes in coping process variables within latent depressive symptoms trajectories

**Fig. 2:**
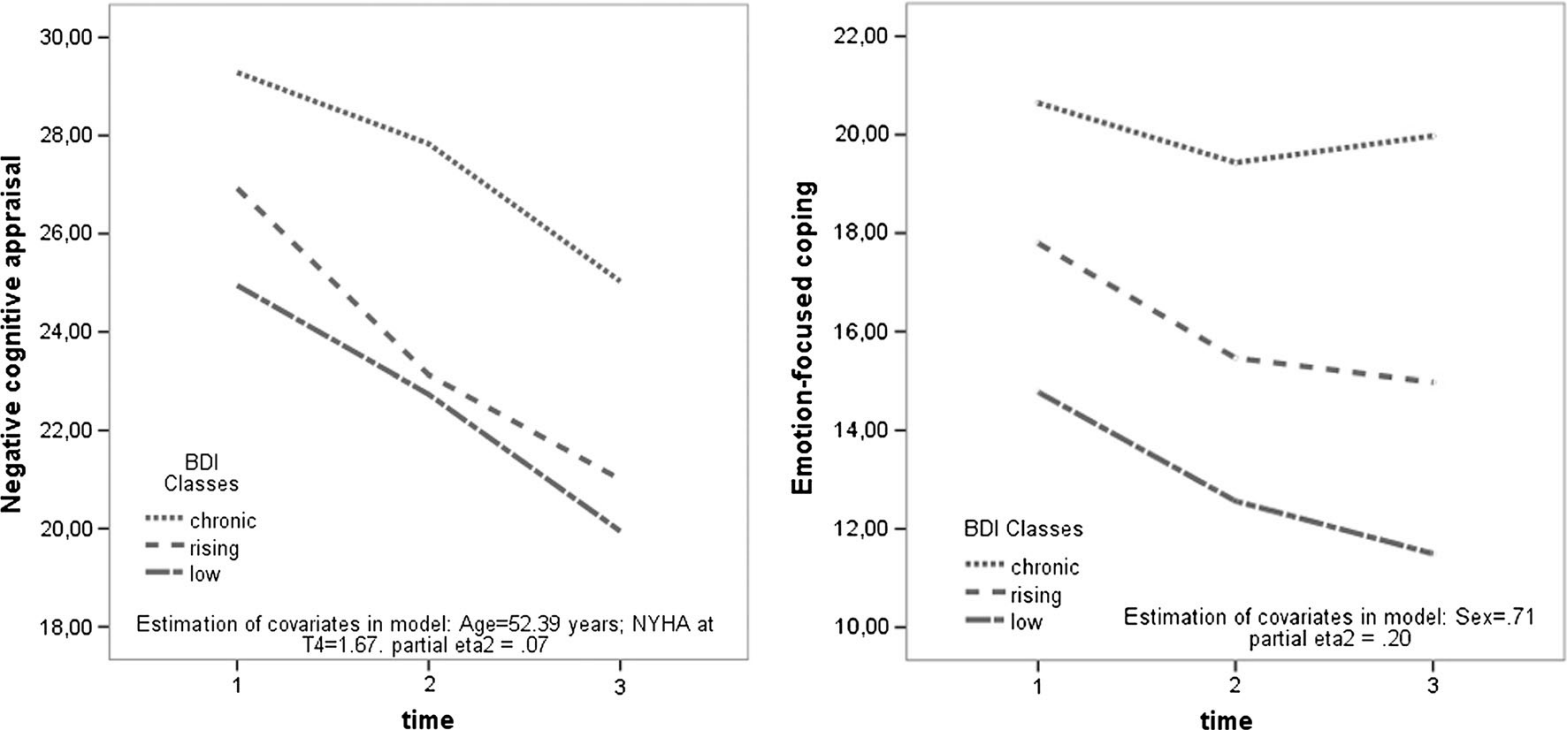
Changes in negative cognitive appraisal and emotion-focused coping during the first 6 months after MI among participants with different depressive symptom trajectories

The 3 (depressive trajectories) × 3 (time) repeated measures MANCOVA showed significant between-subject effects (a main depressive latent class effect) in negative appraisal and emotion-focused coping (see Table 4; Fig. 2). Patients characterized by a chronic depressive trajectory showed a higher level of negative appraisal and emotion-focused coping than those in the rising or low class regardless of time. Furthermore, the comparison for emotion-focused coping between the rising and low subgroups also proved significant. Interaction effects (trajectory × time) were not detected. Within-subject effects (a main time effect) were similar to those shown in Table 1, without covariates.

**Table 4:** Results of multivariate mixed models of covariance–a main depressive latent class effect

	Chronic depression *n* = 49 (1)			Rising depression *n* = 121 (2)			Low depression *n* = 30 (3)			Between-subject effect^a^
	T1	T2	T3	T1	T2	T3	T1	T2	T3	*F* value^b^
M (SD)										
Negative appraisal^c,d^	29.73 (6.66)	28.87 (7.04)	26.30 (8.04)	26.82 (6.96)	23.05 (7.55)	20.78 (8.07)	24.62 (7.63)	21.47 (8.99)	18.76 (7.02)	4.07* (1) > (2), (3)
Positive appraisal	22.37 (3.59)	21.82 (3.85)	21.52 (4.57)	21.58 (3.88)	22.43 (3.84)	22.72 (3.77)	22.03 (4.57)	22.73 (4.52)	21.53 (5.29)	.16
Problem coping	27.67 (5.52)	27.07 (4.84)	25.74 (5.15)	27.44 (5.07)	26.76 (5.22)	25.65 (5.26)	26.95 (6.27)	27.30 (6.17)	25.57 (5.88)	.04
Emotion coping^e^	20.98 (5.69)	19.61 (4.34)	20.06 (4.26)	17.75 (5.90)	15.44 (5.48)	14.97 (5.46)	14.40 (6.25)	12.37 (4.71)	11.40 (5.02)	24.48*** (1) > (2) > (3)
Avoidance coping	15.43 (5.29)	14.56 (4.45)	13.74 (5.19)	14.39 (4.93)	13.90 (4.41)	13.54 (4.17)	14.27 (4.60)	13.30 (5.34)	12.57 (4.82)	.91

* *p* < .05; ** *p* < .01; *** *p* < .001

^a^Pairwise comparisons with Bonferroni’s test. ^b^ *df* = 2;196

Used covariates (only those which turned out to be significant for dependent variables): ^c^ age, ^d^ NYHA in T4, ^e^ sex

## Discussion and conclusions

As can best be determined, this study is the first to examine longitudinal patterns of change in post-MI depressive symptoms over a period of 6 years and the first to examine crucial coping process variables as predictors of post-MI depressive trajectory. In this study, stress and coping theory (Lazarus & Folkman, 1984), and especially the role of cognitive appraisal and coping, were analyzed using a person-centered perspective. The findings revealed heterogeneity in depressive trajectories among MI survivors over a 6-year follow-up period, namely, three different trajectories were found. Only the hypothesis concerning latent change classes in depressive symptoms over time was confirmed (Hypothesis 1b). More than half of MI patients (55.7 %) reported medium symptoms up to 1 month after MI, than decreases 6 months later, and subsequently increases 6 years post-MI (rising subgroup). Participants with the chronic trajectory (29.1 %) demonstrated high symptoms up to 1 month after MI, than increases in the sixth month after MI, and decreases 6 years later. The smallest class (15.2 %) reported relatively low initial levels of depressive symptoms (up to 1 month post-MI) with decreases 6 months later and subsequent stability. It is important to note that, based on the analysis of data from a variable-centered perspective (in the entire sample), participants showed decreases in depressive symptoms 1 month after MI, and then stability, which may indicate adequate adaptation to MI. However, this does not take into consideration about a third of the patients with permanently high symptoms. Thus, the longitudinal person-centered perspective gives support to Lazarus and Folkman (1984) theory and Bonanno (2004) findings of individualized adaptation to stressful events.

For patients with the rising trajectory, adaptation to the cardiac event seems to be adequate over the first 6 months post-MI, then depressive symptoms slightly grew at the 6-year follow-up. The increase in depressive symptoms may result from additional difficult life events following the MI, e.g., loss of employment, subsequent worsening of finances, etc. It is also possible that existing symptoms were exacerbated over time. Alternatively, the worsening might be related to a return to baseline (pre-MI) functioning, particularly as the pre-cardiac event emotional state of the patients was unknown in this study. Therefore, how the various trajectories correspond with patient functioning before the disease cannot be defined. On the other hand, patients followed the chronic trajectory demonstrated permanently high depression with an increase 6 months after MI. It is possible that this subgroup was characterized by generally worse mental health, even before MI, as similarly suggested by a prospective study by Galatzer-Levy and Bonanno (2014).

The trajectory patterns identified in this study are similar to those observed in other illness populations (Bonanno et al., 2012; Chilcot et al., 2013; Donovan et al., 2014; Galatzer-Levy & Bonanno, 2014; Kaptein et al., 2006; Murphy et al., 2008). However, a large majority of the patients had the rising, not low, trajectory of depressive symptoms. Moreover, a group with a significantly improved well-being, usually called the recovery group, was not identified. In previous studies, the trajectory with the fewest depressive symptoms (stable or change—decreasing) usually comprised more than 60 % of the participants (Bonanno et al., 2012; Chilcot et al., 2013; Galatzer-Levy & Bonanno, 2014; Kaptein et al., 2006). It is important to note that the findings of the present and previous studies are not directly comparable because of differences in the length of the follow-up intervals. Findings of this study might have resulted from the group characteristics. Specifically, the sample was dominated by middle-aged retired men, which is typical for this group of patients (Mendis et al., 2011), also in Poland. According to the SHARE Project (Börsch-Supan et al., 2008), this group’s well-being is worse, not only compared with a younger sample from the Polish population, but also compared with Western peers. Perhaps this is why the low trajectory class had so few representatives.

The inclusion in a particular post-MI depressive trajectory group was related to coping process variables. These results support stress and coping theory (Lazarus & Folkman, 1984), albeit as a partially confirmed hypothesis (2a, 2b). Negative appraisal (threat/loss) and emotion-focused coping predicted class membership. Patients in the chronic depression subgroup showed higher negative appraisal and emotion-focused coping than those in the rising or low class regardless of time point. In addition, those in the rising subgroup showed higher emotion-focused strategies than the low class. No effects of positive (challenge) appraisal and either instrumental or avoidance coping were observed. The results are in agreement with mixed effects models of crucial coping variables and stress-outcome trajectories. For example, Bonanno et al. (2012) found a predictive function of challenge appraisal and all coping strategies. In contrast, Lambert et al. (2012) only indicated a differentiating role of avoidance. Donovan et al. (2014) concluded that coping did not predict depressive classes; however, one strategy, “focusing on symptoms” (which can be considered emotion-focused), was associated with higher depressive level. Because in current study all measured aspects focus on negative emotions (depression), beliefs (appraisal) and concentration on these states (emotion-focused coping), the findings are not surprising. A relationship between these variables is usually observed in studies. However, the effects of item contamination should also be considered. The current findings support the general tendency for negativity bias—negativity is more powerful, and thus manifests itself more strongly than positivity (Baumeister et al., 2001). However, more research is required in this area. Specifically, future studies should examine the conditions under which negativity bias occurs, such as by evaluating the effects of challenge appraisal or instrumental coping on depressive symptoms. Nonetheless, it is clear that less concentration on emotions may be the key to resilience after a cardiac event.

Depressive symptoms trajectory classes also differed in certain background and health-related factors. The largest differentiation was noted between the chronic and low subgroups. Participants with fewer symptoms were younger, better educated, employed, and had better socioeconomic positions. This corresponds with well-known risk factors for depression (Doyle et al., 2011). Similar results were noted in prior depression course studies (Chilcot et al., 2013). The significance of somatic health on well-being was also confirmed in the present study. Participants with fewer depressive symptoms were healthier; they assessed their heart failure better, and took less medication. Furthermore, these relationships do not appear to be linear. Further studies are required to clarify the various associations.

In the practical sense, it appears that clinicians should focus on patients with negative cognition and those who focus on negative states. As shown, these states are not conducive to adjustment. Low palliative coping seems to be key to staying resilient. Finally, patients with worse health conditions should not be neglected, because they are a significant risk factor for depression.

The findings have important theoretical and practical implications; however, several limitations should be noted. First, the sample was dominated by poorly-educated, retired men, which limits the generalizability of the results. However, this is the socio-demographic profile of most post-MI patients, also in Poland. Further studies are required to explore the post-MI coping process among women and evaluate gender differences. Second, the three depressive trajectories are not necessarily the only ones that might exist in this patients’ population. There is a probability that analyzing a larger cohort of patients, can reveal other depressive trajectories. Furthermore, in accordance with prior research in this area, present study was limited to examinations of quantitative changes in post-MI depressive symptoms. Changes in depressive symptoms are likely to be qualitative as well. Depressive symptoms comprise cognitive, affective, behavioral, motivational and somatic symptoms, which may vary in different ways over time (World Health Organization, 1993). Moreover, there are indications that post-MI depressive somatic symptoms have an especially harmful effect on patient health (Bekke-Hansen et al., 2012). Therefore, future studies should examine whether there are qualitative differences in longitudinal patterns of post-MI depression. In addition, although the extensive follow-up period was one of the strengths of this study, the time that lapsed between measurements was not equal, and the length between the third and fourth measurement was very long. Therefore, the impact of other possible important factors for patient well-being could not be evaluated, despite controlling for the presence of critical life events at the final timepoint. Accordingly, future studies should use equal time intervals, if possible. Despite the limitations, the findings highlight the diverse nature of emotional adaptation in MI survivors in the long-term and the manner in which cognitive appraisal and coping strategies are related to different longitudinal trajectories of post-MI depressive symptoms. More research is needed in this group using the proposed methodology and analysis in order to generalize the results to the wider MI population. A meta-analysis of such research would allow us to further understand the course of adaptation paths after stressful event and the role of coping in this process.

## Electronic supplementary material

Supplementary material 1 (DOCX 27 kb)
