# Supplementary material for: Depressive symptom trajectories over a 6-year period following myocardial infarction: predictive function of cognitive appraisal and coping
Source: J Behav Med. 2015 Sep 30;39:181–91. doi: 10.1007/s10865-015-9681-y (PMC4799799; doi:10.1007/s10865-015-9681-y)
Supplement: Supplementary file 1 — Supplementary material 1 (DOCX 27 kb) [file 10865_2015_9681_MOESM1_ESM.docx]

SUPPLEMENTARY MATERIAL

Table 1R. Descriptive statistics (*mean* ± *SD*) and test *t*-difference results for original (*N*=111) and imputed (*N*=200) data-sets.

| Variables | Original data  *N*=111 | | Imputed data  *N*=200 | | *t*-difference | *p* |
| --- | --- | --- | --- | --- | --- | --- |
|  | *M* | *SD* | *M* | *SD* |  |  |
| Depressive symptoms T1 | 9.3727 | 7.15088 | 10.5700 | 8.60379 | 1.314 | .904 |
| Depressive symptoms T2 | 8.0091 | 6.73958 | 8.8824 | 7.13228 | 1.064 | .856 |
| Depressive symptoms T3 | 7.5909 | 7.44558 | 7.9194 | 7.48430 | 0.373 | .645 |
| Depressive symptoms T4 | 8.0818 | 6.78792 | 8.4924 | 6.49009 | 0.517 | .697 |
| Negative appraisal T1 | 27.2202 | 7.20128 | 28.2212 | 7.20956 | 1.169 | .878 |
| Negative appraisal T2 | 24.4273 | 8.11213 | 25.3409 | 7.77797 | .959 | .831 |
| Negative appraisal T3 | 21.9818 | 8.27507 | 22.8450 | 8.46750 | .869 | .807 |
| Positive appraisal T1 | 21.6667 | 3.97186 | 21.8665 | 3.92819 | .426 | .664 |
| Positive appraisal T2 | 22.2364 | 3.89761 | 22.3101 | 3.75551 | -.066 | .474 |
| Positive appraisal T3 | 22.0909 | 4.40714 | 22.2302 | 4.02114 | .276 | .608 |
| Problem coping T1 | 26.8879 | 5.03821 | 27.4270 | 5.34926 | .883 | .811 |
| Problem coping T2 | 26.3182 | 5.59477 | 26.9149 | 5.26016 | .908 | .817 |
| Problem coping T3 | 25.0364 | 5.54701 | 25.6600 | 5.30626 | .956 | .823 |
| Emotion coping T1 | 17.4091 | 5.76496 | 18.0400 | 6.21785 | .895 | .814 |
| Emotion coping T2 | 15.4091 | 5.69290 | 16.0187 | 5.61126 | .908 | .812 |
| Emotion coping T3 | 15.2818 | 5.78218 | 15.7047 | 5.82638 | .61 | .728 |
| Avoidance coping T1 | 14.1193 | 5.10034 | 14.6280 | 4.97253 | .85 | .802 |
| Avoidance coping T2 | 13.3727 | 4.85356 | 13.9655 | 4.56264 | 1.047 | .851 |
| Avoidance coping T3 | 12.3818 | 4.50073 | 13.4196 | 4.54051 | 1.941 | .973 |

Comparisons were made using calculator from http://www.quantitativeskills.com/sisa/statistics.

Table 2R. Correlations coefficients (*r*-Pearson) between analyzed variables of original (*N*=111; lower diagonal) and imputed (*N*=200; upper diagonal) data.

|  | **1.** | **2.** | **3.** | **4.** | **5.** | **6.** | **7.** | **8.** | **9.** | **10.** | **11.** | **12.** | **13.** | **14.** | **15.** | **16.** | **17.** | **18.** | **19.** |
| --- | --- | --- | --- | --- | --- | --- | --- | --- | --- | --- | --- | --- | --- | --- | --- | --- | --- | --- | --- |
| 1. BDI_1 | 1 | ,699^**^ | ,654^**^ | ,688^**^ | ,494^**^ | ,410^**^ | ,358^**^ | -,016 | -,066 | -,069 | -,008 | ,070 | ,053 | ,485^**^ | ,526^**^ | ,427^**^ | ,075 | ,093 | ,120 |
| 2. BDI_2 | ,726^**^ | 1 | ,751^**^ | ,723^**^ | ,417^**^ | ,495^**^ | ,398^**^ | ,047 | -,127 | -,103 | ,055 | ,094 | ,082 | ,499^**^ | ,632^**^ | ,473^**^ | ,191^**^ | ,242^**^ | ,246^**^ |
| 3. BDI_3 | ,630^**^ | ,755^**^ | 1 | ,774^**^ | ,326^**^ | ,430^**^ | ,436^**^ | ,081 | -,095 | -,116 | ,117 | ,104 | ,078 | ,407^**^ | ,519^**^ | ,522^**^ | ,131 | ,138 | ,099 |
| 4. BDI_4 | ,556^**^ | ,579^**^ | ,660^**^ | 1 | ,349^**^ | ,437^**^ | ,406^**^ | ,045 | -,122 | -,119 | ,078 | ,102 | ,075 | ,428^**^ | ,522^**^ | ,515^**^ | ,144^*^ | ,134 | ,152^*^ |
| 5. Neg App_1 | ,506^**^ | ,409^**^ | ,317^**^ | ,283^**^ | 1 | ,646^**^ | ,526^**^ | ,183^**^ | ,121 | ,122 | ,200^**^ | ,319^**^ | ,224^**^ | ,539^**^ | ,535^**^ | ,434^**^ | ,324^**^ | ,259^**^ | ,209^**^ |
| 6. Neg App_2 | ,452^**^ | ,494^**^ | ,431^**^ | ,403^**^ | ,624^**^ | 1 | ,640^**^ | ,134 | ,125 | ,051 | ,193^**^ | ,255^**^ | ,253^**^ | ,411^**^ | ,586^**^ | ,517^**^ | ,307^**^ | ,286^**^ | ,327^**^ |
| 7. Neg App_3 | ,433^**^ | ,488^**^ | ,447^**^ | ,424^**^ | ,531^**^ | ,587^**^ | 1 | ,052 | ,013 | ,115 | ,138 | ,233^**^ | ,274^**^ | ,327^**^ | ,521^**^ | ,587^**^ | ,266^**^ | ,101 | ,258^**^ |
| 8. Pos App_1 | ,100 | ,103 | ,130 | ,109 | ,166 | ,064 | -,050 | 1 | ,460^**^ | ,218^**^ | ,310^**^ | ,152^*^ | ,206^**^ | ,138 | ,058 | ,037 | ,100 | ,089 | ,064 |
| 9. Pos App_2 | ,042 | -,169 | -,192^*^ | -,160 | ,071 | -,006 | -,178 | ,415^**^ | 1 | ,536^**^ | ,267^**^ | ,377^**^ | ,181^*^ | ,210^**^ | ,012 | ,111 | ,051 | ,055 | ,057 |
| 10. Pos App_3 | -,008 | -,158 | -,146 | -,138 | ,088 | -,053 | ,037 | ,175 | ,521^**^ | 1 | ,154^*^ | ,251^**^ | ,276^**^ | ,145^*^ | ,055 | ,186^**^ | ,070 | ,093 | ,146^*^ |
| 11. Problem_1 | -,052 | ,007 | ,119 | ,063 | ,169 | ,096 | ,009 | ,289^**^ | ,103 | ,128 | 1 | ,476^**^ | ,569^**^ | ,367^**^ | ,152^*^ | ,259^**^ | ,352^**^ | ,306^**^ | ,188^**^ |
| 12. Problem_2 | ,149 | ,105 | ,115 | ,128 | ,338^**^ | ,249^**^ | ,227^*^ | ,120 | ,354^**^ | ,208^*^ | ,488^**^ | 1 | ,548^**^ | ,220^**^ | ,261^**^ | ,278^**^ | ,193^**^ | ,328^**^ | ,258^**^ |
| 13. Problem_3 | ,090 | ,052 | ,075 | ,073 | ,248^**^ | ,251^**^ | ,235^*^ | ,158 | ,157 | ,244^*^ | ,602^**^ | ,532^**^ | 1 | ,222^**^ | ,187^**^ | ,373^**^ | ,267^**^ | ,180^*^ | ,326^**^ |
| 14. Emotion_1 | ,503^**^ | ,437^**^ | ,385^**^ | ,341^**^ | ,508^**^ | ,295^**^ | ,220^*^ | ,108 | ,148 | ,168 | ,290^**^ | ,214^*^ | ,274^**^ | 1 | ,615^**^ | ,569^**^ | ,250^**^ | ,252^**^ | ,155^*^ |
| 15. Emotion_2 | ,579^**^ | ,663^**^ | ,563^**^ | ,488^**^ | ,535^**^ | ,570^**^ | ,501^**^ | ,025 | -,060 | -,004 | ,061 | ,276^**^ | ,174 | ,556^**^ | 1 | ,707^**^ | ,204^**^ | ,279^**^ | ,208^**^ |
| 16. Emotion_3 | ,478^**^ | ,484^**^ | ,549^**^ | ,524^**^ | ,405^**^ | ,430^**^ | ,472^**^ | -,038 | ,001 | ,120 | ,190^*^ | ,281^**^ | ,333^**^ | ,527^**^ | ,686^**^ | 1 | ,217^**^ | ,189^**^ | ,244^**^ |
| 17. Avoid_1 | ,032 | ,131 | ,110 | ,106 | ,321^**^ | ,309^**^ | ,314^**^ | ,065 | -,060 | ,056 | ,294^**^ | ,206^*^ | ,249^**^ | ,168 | ,283^**^ | ,200^*^ | 1 | ,497^**^ | ,588^**^ |
| 18. Avoid_2 | -,005 | ,129 | ,081 | ,034 | ,170 | ,290^**^ | ,099 | ,011 | -,005 | ,090 | ,200^*^ | ,216^*^ | ,073 | ,145 | ,275^**^ | ,149 | ,520^**^ | 1 | ,600^**^ |
| 19. Avoid_3 | ,110 | ,175 | ,052 | ,107 | ,153 | ,275^**^ | ,252^**^ | -,047 | -,069 | ,090 | ,053 | ,151 | ,171 | ,095 | ,253^**^ | ,176 | ,574^**^ | ,559^**^ | 1 |

Note: **p*<.05; ***p*<.01.

Table 3R. Mean and standard deviations of affective and somatic depression symptoms (T1-T4) by latent trajectories. Results of ANOVA.

|  | Chronic class  *n*=49 | | Rising class  *n*=121 | | Low class  *n*=30 | | ANOVA | |
| --- | --- | --- | --- | --- | --- | --- | --- | --- |
|  | *M* | *SD* | *M* | *SD* | *M* | *SD* | *F* | *p* |
| Affective T1 | 6.9592 | 7.16752 | 5.9339 | 5.68439 | 6.6000 | 5.10308 | .557 | .574 |
| Affective T2 | 6.0513 | 5.88016 | 4.5893 | 4.72210 | 3.5172 | 3.58191 | 2.410 | .093 |
| Affective T3 | 6.3514 | 7.23885 | 3.9091 | 4.62055 | 3.0769 | 3.40497 | 2.849 | .066 |
| Affective T4 | 5.5517 | 5.85288 | 3.9254 | 3.75505 | 4.6429 | 5.69220 | .972 | .390 |
| Somatic T1 | 4.2653 | 3.98840 | 4.3140 | 3.12259 | 4.2000 | 3.20990 | .015 | .985 |
| Somatic T2 | 4.2308 | 2.99527 | 4.2321 | 3.21870 | 3.4483 | 2.81052 | .772 | .464 |
| Somatic T3 | 4.1053 | 3.43109 | 3.4818 | 3.07022 | 2.9231 | 2.97890 | 1.137 | .323 |
| Somatic T4 | 3.7931 | 2.56876 | 3.5147 | 2.79921 | 3.6429 | 3.27243 | .102 | .903 |
